# Antiviral activities of hemp cannabinoids

**DOI:** 10.1042/CS20220193

**Published:** 2023-04-21

**Authors:** Richard B. van Breemen, Daniel Simchuk

**Affiliations:** Department of Pharmaceutical Sciences, College of Pharmacy, Linus Pauling Institute, Global Hemp Innovation Center, Oregon State University, 2900 SW Campus Drive, Corvallis, OR 97331, U.S.A.

**Keywords:** antiviral, cannabinoids, COVID-19, herpesvirus, HIV, SARS-CoV-2

## Abstract

Hemp is an understudied source of pharmacologically active compounds and many unique plant secondary metabolites including more than 100 cannabinoids. After years of legal restriction, research on hemp has recently demonstrated antiviral activities *in silico*, *in vitro*, and *in vivo* for cannabidiol (CBD), Δ^9^-tetrahydrocannabinol (Δ^9^-THC), cannabidiolic acid (CBDA), cannabigerolic acid (CBGA), and several other cannabinoids against severe acute respiratory syndrome coronavirus-2 (SARS-CoV-2), human immunodeficiency virus (HIV), and γ-herpes viruses. Mechanisms of action include inhibition of viral cell entry, inhibition of viral proteases, and stimulation of cellular innate immune responses. The anti-inflammatory properties of cannabinoids are also under investigation for mitigating the cytokine storm of COVID-19 and controlling chronic inflammation in people living with HIV. Retrospective clinical studies support antiviral activities of CBD, Δ^9^-THC, and cannabinoid mixtures as do some prospective clinical trials, but appropriately designed clinical trials of safety and efficacy of antiviral cannabinoids are urgently needed.

## Introduction

Hemp (*Cannabis sativa* L.) has been cultivated for millennia as a source of fiber, food, and medicine [1,2]. In the United States, industrial hemp was grown as a commodity fiber crop from the mid-18th century until the mid-1930s. From 1936 until 2014, propagation of hemp plants was prohibited in the United States, but this changed as the federal government began to permit the commercial production and utilization of hemp for purposes such as essential oils and fiber production.

**Figure 1:**
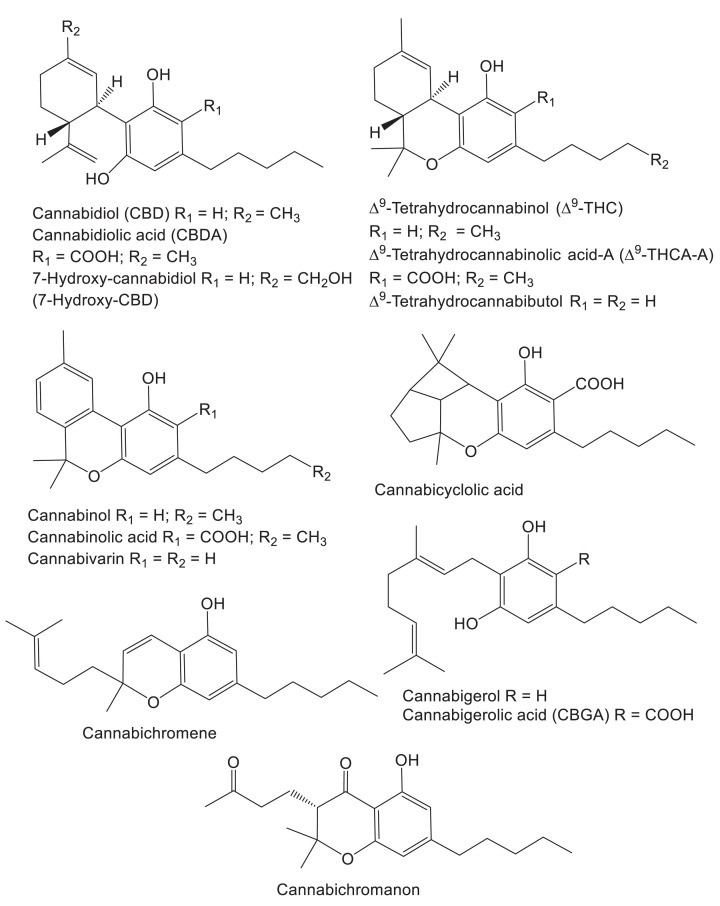
Cannabinoids produced by hemp with antiviral and other therapeutic activities

In addition to fiber and essential oils, the hemp plant produces hundreds of secondary metabolites including flavonoids, diterpenes, triterpenes, and cannabinoids [3,4]. Unlike primary metabolites such as proteins, nucleic acids, lipids, and carbohydrates that are essential for life, secondary metabolites benefit plants in other ways such as deterring predators, attracting pollinators, or preventing infection. Several secondary metabolites that are unique to *Cannabis sativa* L. include some cannflavins (flavonoids), cannabisins (lignans), and cannabinoids. Hemp produces over 100 cannabinoids including cannabidiol (CBD), cannabidiolic acid (CBDA), the psychotropic cannabinoid Δ^9^-tetrahydrocannabinol (Δ^9^-THC), Δ^9^-tetrahydrocannabinolic acid-A (Δ^9^-THCA-A), cannabigerol, cannabigerolic acid (CBGA), and Δ^9^-tetrahydrocannabutol (Figure 1) [5,6].

Synthetic Δ^9^-THC was approved by the United States Food and Drug Administration in 1985 as the drug dronabinol for the treatment of nausea associated with cancer chemotherapy and for anorexia associated with weight loss in AIDS patients [7]. The synthetic Δ^9^-THC analog nabilone has been approved in the United States for the control of chemotherapy-induced nausea and in Canada as an adjunct therapy for chronic pain management [8]. Discovered in 1940, CBD (Figure 1) lacks the psychotropic properties of Δ^9^-THC but exhibits other activities including some with established therapeutic benefit. In 2018, CBD isolated from hemp was approved in the United States as the drug epidiolex for the treatment of epileptic seizures known as Lennox–Gastaut syndrome and Dravet syndrome [9].

Despite legal restrictions, cannabinoids and other compounds from hemp have a long and extensive history of safe use in humans. These products have been administered orally, sublingually, dermally, and by inhalation. As a class of natural products, cannabinoids have been shown to have suitable oral bioavailability, metabolism, blood–brain barrier permeability, and safety for use as therapeutic agents [10,11]. Based on the established therapeutic efficacy of CBD and Δ^9^-THC, these and other cannabinoids are under investigation for additional pharmacological activities, including activity as antiviral agents.

Inspired by the success of penicillin in treating certain bacterial infections, intense antibiotic drug discovery research was carried out by the pharmaceutical industry from 1945 until the mid-1960s. Similar efforts to discover antiviral agents during this time were disappointing, and most active antiviral agents like idoxuridine were highly toxic if used systemically [12]. Regarding antiviral drugs to be inherently toxic to the human host, most antiviral drug discovery efforts were abandoned until Hitchings, Elion, and Schaeffer discovered acyclovir in 1974 [13]. By targeting unique viral enzymes and pathways uncovered through the application of molecular biology, acyclovir and related nucleoside analog drugs were shown to be highly effective against herpes viruses while low in toxicity [14].

## Antiviral cannabinoids

### Δ^9^-THC

**Figure 2:**
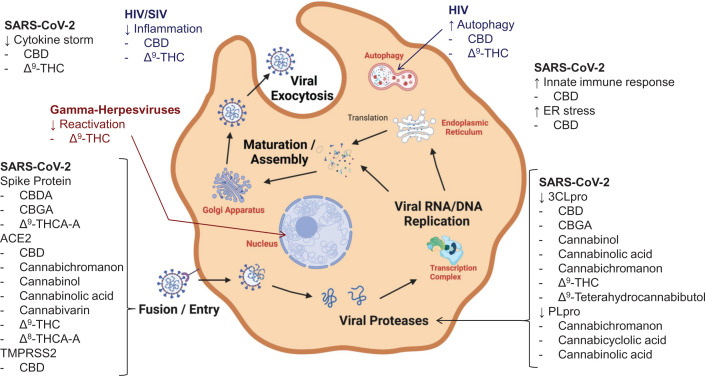
Antiviral targets of cannabinoids Most antiviral cannabinoids inhibit cell entry, inhibit viral proteases, or prevent viral induced inflammation.

One of the earliest reports of antiviral activity of cannabinoids was the discovery in 2004 that Δ^9^-THC could prevent the activation of the viral genome of latent gamma-herpesviruses in cell culture (Table 1 and Figure 2) [15]. Micromolar concentrations of Δ^9^-THC, which were not cytotoxic, inhibited Kaposi's sarcoma-associated herpesvirus (IC_50_ 3.3 μM) and Epstein-Barr (IC_50_ 3.0 μM) virus reactivation in infected immortalized B cells. Δ^9^-THC also strongly inhibited lytic replication of herpesvirus saimiri of monkeys and murine gamma-herpesvirus *in vitro*. The mechanism of action was proposed to be suppression of transcription factors that activate the promoter of the critical viral gene open reading frame of Kaposi’s sarcoma-associated herpesvirus or its homologue Rta of Epstein–Barr virus, which activate early lytic genes involved in virus DNA synthesis.

**Table 1:** Antiviral cannabinoids and mechanisms of action

Compound	Virus	Target/Mechanism of Action	Reference
CBD	HIV	Up-regulation of autophagy receptor p62/SQSTM1 reduces viral spread in human monocytic THP-1 cells and primary human macrophages	Tomer et al., 2022 [39]
	SARS-CoV-2	Inhibits viral replication in Vero cells (IC_50_ = 7.91 μM)	Raj et al., 2021 [21]
		Downregulates expression of ACE2 in human cells	Anil et al., 2021 [25]
		Inhibits main protease 3CLpro (IC_50_ = 1.86 μM)	Pitakbut et al., 2022 [22]
		Inhibits human ACE2 (IC_50_ = 14.65)	Pitakbut et al., 2022 [22]
		Downregulates expression of TMPRSS2 on human cells, and blocks induction of inflammatory mediators via the AKT pathway	Wang et al., 2022 [26]
		Inhibits viral replication in A549-ACE2 human lung carcinoma cells (EC_50_ = 1.24 μM)	Nguyen et al., 2022 [27]
		Stresses the endoplasmic reticulum of the host cell, stimulates innate immune responses, and inhibits the stimulation of cytokines associated with COVID-19	Nguyen et al., 2022 [27]
		In a retrospective study, negative association between patients taking CBD for control of epileptic seizures and SARS-CoV-2 infection	Nguyen et al., 2022 [27]
		In a prospective clinical trial, no benefit of 300 mg/day per oral CBD for 14 days in mild to moderate COVID-19 patients	Crippa et al., 2022 [30]
		Downregulates ACE2, TMPRSS2, and COX-2 and suppresses release of IL-6 and IL-8 by AKT pathway in lung fibroblast WI-38 cells	Wang et al., 2022 [26]
		CBD (5 mg/kg) reduces IL-6 and TNF-α and lowers lung infiltration by macrophages and neutrophils in mice with acute respiratory distress syndrome resembling COVID-19	Khodadadi et al., 2020 [33]
		Inhibits LPS-induced inflammatory responses in THP-1 macrophages and primary human bronchial epithelial cells as a model of the COVID-19 cytokine storm	Suryavanshi et al., 2022 [35]
CBD + Terpenes	SARS-CoV-2	Terpene extract potentiates CBD inhibition of viral replication	Santos et al., 2022 [36]
7-Hydroxy-CBD	SARS-CoV-2	Inhibits viral replication in A549-ACE2 cells (EC_50_ = 3.60 μM)	Nguyen et al., 2022 [27]
CBDA	SARS-CoV-2	Binds orthosterically to the SARS-CoV-2 spike protein S1 subunit (*K*_d_ = 19.8 μM) and prevents infection of human epithelial cells and Vero cells by SARS-CoV-2 and early variants	van Breemen et al., 2022 [41]
CBGA	SARS-CoV-2	Binds orthosterically and allosterically to the SARS-CoV-2 spike protein S1 subunit (*K*_d_ = 5.6 μM) and prevents cell entry of human epithelial cells and Vero cells by SARS-CoV-2 and early variants	van Breemen et al., 2022 [41]
		Inhibits the viral protease 3CLpro (IC_50_ = 14.40 μM)	Liu et al., 2022 [23]
Δ^9^-THCA-A	SARS-CoV-2	Binds orthosterically to the SARS-CoV-2 spike protein S1 subunit	van Breemen et al., 2022 [41]
Cannabivarin and Δ^8^-THCA-A	SARS-CoV-2	Molecular modeling predicts inhibition of human ACE2	El Ouafi et al., 2022 [43]
Cannabichromanon and cannabinolic acid	SARS-CoV-2	Molecular docking predicts inhibition of viral proteases 3CLpro and PLpro and human ACE2	Altyar et al., 2022 [42]
Cannabinol	SARS-CoV-2	Molecular docking predicts inhibition of 3CLpro and ACE2	Altyar et al., 2022 [42]
Cannabicyclolic acid	SARS-CoV-2	Molecular docking predicts inhibition of viral protease PLpro	Altyar et al., 2022 [42]
Δ^9^-THC	gamma-Herpes Viruses	Inhibits viral replication; inhibits KSHV and EBV reactivation in infected immortalized B cells	Medveczky et al., 2004 [15]
	SARS-CoV-2	Inhibits viral replication in Vero cells (IC_50_ = 10.25 μM)	Raj et al., 2021 [21]
		Inhibits viral protease 3CLpro (IC_50_ = 16.23 μM)	Pitakbut et al., 2022 [22]
		Inhibits human ACE2 (IC_50_ = 11.47 μM)	Pitakbut et al., 2022 [22]
		Inhibits LPS-induced cytokine storm in human macrophages and primary bronchial epithelial cells by modulating NLRP3 inflammasome and STAT3 signaling	Suryavanshi et al., 2022 [35]
	SIV	Reduces viral replication and intestinal inflammation and slows disease progress in rhesus macaques	Molina et al., 2011 [16] Chandra et al., 2015 [17]
		Counteracts proinflammatory responses in rhesus macaques	Kaddour et al., 2022 [18]
Δ^9^-Tetrahydro- cannabibutol	SARS-CoV-2	Inhibits viral 3CLpro (IC_50_ 3.62 µM)	Liu et al., 2022 [23]
*Cannabis sativa*	HIV	Clinical study indicating heavy cannabis use reduces inflammation associated with HIV	Manuzak et al., 2018 [19]
	SARS-CoV-2	Reduces expression of ACE2 and inflammatory cytokines including IL-6 and IL-8 in A549 human alveolar epithelial cells	Anil et al., 2021 [25]
		In a retrospective study, cannabis improves clinical outcomes and lowers inflammatory markers in hospitalized COVID-19 patients	Shover et al., 2022 [29]
		Hemp inflorescence extract improves symptoms of post COVID conditions (long-COVID)	Young et al., 2022 [32]
		Intravenous high-CBD hemp extract reduces lung inflammation, systemic inflammation, and neutrophil migration to the lungs in mice	Aswad et al., 2022 [34]

Using rhesus macaques infected with simian immunodeficiency virus (SIV) as a model of HIV, Molina et al. [16] found that chronic intramuscular administration of Δ^9^-THC (0.32 mg/kg, twice daily) decreased plasma and cerebral spinal fluid viral load, maintained body mass, and decreased mortality due to viral infection (Table 1). Using MT4-R5 cells in culture, they also reported that 10 μm Δ^9^-THC decreased SIV replication in MT4-R5 cells. In 2015, this same group confirmed that Δ^9^-THC inhibited replication of this virus and slowed disease progression in infected rhesus macaques [17]. They also found that Δ^9^-THC protected against infection-induced gastrointestinal inflammation by up-regulating anti-inflammatory miRNAs in the duodenum. In a 2022 follow-up study, this group reported that chronic administration of low dose Δ^9^-THC counteracted proinflammatory effects of SIV in macaques (Figure 2) [18]. Neuroprotective and anti-inflammatory factors induced by Δ^9^-THC were found to be mediated by basal ganglia-extracellular vesicles-associated microRNAs. These microRNAs corresponded to genes related to inflammation, immune regulation, Toll-like receptor signaling, and cell death.

In 2018, Manuzak et al. [19] investigated possible effects of cannabis use in 198 HIV-infected participants receiving antiretroviral therapy. Based on plasma levels of the Δ^9^-THC metabolite 11-nor-carboxy-tetrahydrocannabinol [20], 14 participants were classified as heavy cannabis users and 40 participants as moderate users. Compared with non-cannabis users, heavy cannabis users had decreased frequencies of T-cells suggesting that cannabinoids might have immunological benefits. Heavy cannabis users also had decreased frequencies of intermediate and non-classical monocyte subsets, as well as decreased frequencies of interleukin (IL)-23 and tumor necrosis factor-α (TNF-α)-producing antigen-presenting cells, which were indications of reduced systemic inflammation (Table 1 and Figure 2).

In 2021, Raj et al. [21] used molecular docking to screen 32 hemp cannabinoids for binding to 3CLpro, which is the main protease of the severe acute respiratory syndrome coronavirus-2 (SARS-CoV-2) that causes COVID-19. The cannabinoids showing the most favorable binding energies (approximately −10 kcal each) were Δ^9^-THC, Δ^9^-THCA-A, CBD, CBDA, and cannabinol (Figure 1). The antiviral activities of these cannabinoids were then assayed using SARS-CoV-2 in African green monkey Vero cell culture, and Δ^9^-THC was the second most potent cannabinoid after CBD with an IC_50_ value of 10.25 μM for the inhibition of viral replication (Table 1). This antiviral activity was comparable to the drugs lopinavir, chloroquine, and remdesivir (IC_50_ values 8.16–13.15 μM) in the same assay. Using a functional enzyme assay, Pitakbut et al. [22] confirmed that inhibition of 3CLpro was an important mechanism of action by determining that Δ^9^-THC inhibited the proteolytic activity of 3CLpro with an IC_50_ value of 16.23 ± 1.71 μM (Table 1 and Figure 2).

Based on the inhibition of 3CLpro by some of the major cannabinoids like Δ^9^-THC, Liu et al. [23] tested a library of minor cannabinoids for enzymatic inhibition *in vitro*. The most potent inhibitor in this library was Δ^9^- tetrahydrocannabibutol with an IC_50_ value of 3.62 µM. Δ^9^-Tetrahydrocannabibutol is an analog of Δ^9^-THC that contains a butyl side chain instead of a pentyl group (Figure 1). A preliminary structure and activity relationship suggested that the length of the alkyl side chain of cannabinoids influenced activity. For example, shortening the alkyl chain of Δ^9^-THC from a pentyl to a butyl group to form Δ^9^-tetrahydrocannabibutol increased inhibition of 3CLpro by approximately 4-fold.

In addition to inhibiting the SARS-CoV-2 protease 3CLpro, Pikabut et al. [22] found that Δ^9^-THC can inhibit human angiotensin-converting enzyme2 (ACE2), which is an enzyme on the surface of human epithelial cells. Binding of the SARS-CoV-2 spike protein to human ACE2 is an important first step in the infection of human cells. COVID-19 patients receiving ACE2 inhibitors have been found to be at lower risk of mortality and severe adverse events [24]. Δ^9^-THC was shown to inhibit ACE2 with an IC_50_ value of 11.47 ± 3.60 μM (Table 1 and Figure 2) [22].

### CBD

Based on molecular docking studies of the binding of 32 cannabinoids to the SARS-CoV-2 protease 3CLpro followed by antiviral assays using Vero cells, Raj et al. [21] identified CBD as the most potent anti-SARS-CoV-2 among these compounds and that it inhibited virus replication with an IC_50_ value of 7.91 μM (Table 1). Inhibition of SARS-CoV-2 3CLpro was determined to be a mechanism of action of CBD by Pitakbut et al. [22] who reported that CBD inhibited 3CLpro with an IC_50_ value of 1.86 ± 0.04 μM (Table 1). In addition to inhibition of 3CLpro, Pitakbut et al. [22] reported that CBD can inhibit human ACE2 with an IC_50_ value of 14.65 ± 0.47 μM. CBD and hemp extracts rich in CBD can also reduce the expression of ACE2 [25] and TMPRSS2 [26] (both of which are human cell surface receptors that facilitate infection by SAR-CoV-2), and block induction of inflammatory mediators cyclooxygenase-2, IL-6, and IL-8 via the protein kinase B (AKT) pathway [25,26] (Figure 2).

In 2022, Nguyen et al. [27] reported that CBD can inhibit SARS-CoV-2 replication in A549 human lung carcinoma cells expressing the human ACE2 receptor. The effective concentration (EC_50_) of CBD in this assay was 1.24 µM, which was non-toxic to the cells (Table 1). An abundant Phase 1 metabolite of CBD in humans, 7-hydroxy-CBD (Figure 1), was also found to inhibit viral replication in cell culture with an EC_50_ value of 3.60 µM. In a mouse model expressing human ACE2 (K18-hACE2), CBD treatment inhibited SARS-CoV-2 replication in a dose-dependent manner. Based on changes in mRNA levels caused by CBD in cell culture, Nguyen et al. [27] concluded that anti-SARS-CoV-2 mechanisms of action might include inducing stress in the endoplasmic reticulum of the host cell as well as stimulating innate immune responses (Table 1 and Figure 2). Unlike Pitakbut et al. [22], they did not identify inhibition of SARS-CoV-2 3CLpro as a target for CBD.

In a retrospective analysis of hospital records, Nguyen et al. [27] found a negative association between patients taking CBD for control of epileptic seizures and positive diagnosis of SARS-CoV-2 infection (Table 1). They also found that patients reporting frequent use of medicinal or recreational marijuana had a lower risk of COVID-19. Note that consumption of 1500 mg daily of the CBD drug epidiolex has been reported to produce CBD concentrations as high as 1.7 µM [28], which is equivalent to the IC_50_ value of CBD for the inhibition of viral 3CLpro [22].

In another retrospective clinical study, Shover et al. [29] analyzed clinical outcomes of 1831 patients who had been hospitalized in Southern California for COVID-19. Active cannabis users (*n*=69) had significantly better outcomes than non-users including a lower NIH COVID-19 Severity Score (5.1 vs 6.0, *P*<0.001), reduced need for supplemental oxygen, reduced intensive care unit admission (12% vs 31%, *P*<0.001), less need for mechanical ventilation (6% vs 17%, *P*=0.027), and shorter length of hospitalization (4 days vs 6 days, *P*<0.001) (Table 1). Cannabis users hospitalized for COVID-19 also had lower levels of inflammatory markers, although their overall survival rate was not statistically different from non-users. The authors acknowledged that conclusions from retrospective studies need to be interpreted with caution and that prospective studies on the anti-COVID-19 benefits of cannabis are needed.

In the first prospective clinical trial of CBD as a treatment for COVID-19, Crippa et al. [30] evaluated the safety and efficacy of CBD (300 mg/day for 14 days) for treating patients with mild-to-moderate COVID-19. Using a randomized, double-blind design, participants diagnosed with mild-to-moderate COVID-19 receiving the standard of care were randomized to either CBD (*n*=49) or placebo (*n*=42). With respect to safety, CBD was well tolerated, no adverse events were reported, and the side effects of CBD and placebo were indistinguishable. Based on the primary outcomes of recovery time and prevention of worsening clinical status, there were no significant differences between the CBD and placebo treatment groups (Table 1). The authors concluded that further trials are needed to evaluate the efficacy of higher doses of CBD and that efficacy in patients with severe COVID-19 should be investigated. The design of this clinical trial was criticized by Vitella et al. [31] who suggested that because plasma levels only ranged from 10.2 to 17.1 nM, the dosages were too low to be effective. They also suggested that sublingual administration might have produced higher plasma levels than per oral dosing, and that the high percentage of participants with comorbidities (35%), co-administered pharmaceuticals (18%), and alcohol abuse (24%) might have adversely affected intestinal absorption.

In a 28-day randomized, single-blind clinical trial of the safety and efficacy of cannabinoids on post COVID conditions (also known as long-COVID), Young et al. [32] tested a hemp inflorescence extract supplemented with terpenes. Instead of a placebo, the hemp extract was compared inadvertently with a cannabinoid-free mixture of terpenes. The initial dosage of both products was 0.25 ml/day but was titrated up thereafter to optimize effect. The hemp extract contained 40.7 mg/ml CBD, 2.91 mg/ml Δ^9^-THC, 2.28 mg/ml cannabichromene, 3.63 mg/ml CBDA, and 0.94 mg/ml cannabigerol (Figure 1). Out of 23 participants completing the study, 11 participants received the cannabinoid-rich extract while 12 participants received a terpene product. Post COVID conditions improved equally in both treatment groups (Table 1). After 28 days, the study was extended to 70 days in an open label format in which all participants received the study extract containing cannabinoids. No adverse events were reported. The authors acknowledged the omission of a placebo and the small study size as limitations.

In 2020, Khodadadi et al. [33] demonstrated anti-inflammatory effects of CBD in mice with acute respiratory distress syndrome resembling COVID-19 (Table 1). Acute respiratory distress syndrome was induced by intranasal administration of polyinosinic-polycytidylic acid, which stimulates the Toll-like receptor 3. Treatment with CBD (5 mg/kg on days 1, 3, and 5) reduced the levels of the proinflammatory cytokines IL-6 and TNF-α as well as lowered lung infiltration by macrophages and neutrophils.

In a similar study using mice treated with lipopolysaccharide (LPS) intranasally to induce lung inflammation resembling COVID-19 or intraperitoneally to cause systemic inflammation, Aswad et al. [34] found that a high-CBD hemp extract (35% CBD, 0.3% Δ^9^-THC, and 0.3% cannabigerol) reduced inflammation (Table 1). In the lung inflammation model, intravenously injected high-CBD hemp extract (100-150 mg/kg) reduced neutrophil migration to the lungs and decreased IL-1β, IL-6, TNF-α, and monocyte chemoattractant protein-1. In another experiment using mice with systemic inflammation, the high-CBD extract injected intravenously reduced the proinflammatory cytokines IL-6 and TNF-α while increasing the anti-inflammatory cytokine IL-10. Note that Aswad et al. [34] selected this high-CBD extract for evaluation after screening several high-CBD and high-Δ^9^-THC extracts for suppression of IL-6 secretion in LPS-stimulated RAW 264.7 mouse macrophages.

Using human THP-1 macrophages and primary human bronchial epithelial cells, Suryavanshi et al. [35] showed that CBD as well as Δ^9^-THC could inhibit LPS-induced inflammatory responses (Table 1 and Figure 2). Specifically, treatment of cells with CBD or Δ^9^-THC reduced levels of the pro-inflammatory cytokines IL-1β, IL-6, IL-8, and TNF-α. The mechanism of action was found to be inhibition of activation of the nucleotide-binding and oligomerization domain (NOD)-like receptor family pyrin domain-containing 3 inflammasome, and down-regulation of phosphorylation of STAT3. In addition, CBD attenuated phosphorylation of nuclear factor-κB, and both CBD and Δ^9^-THC prevented LPS-induced oxidative stress. The anti-inflammatory activities of CBD and Δ-^9^THC were discussed as of potential benefit to COVID-19 patients.

Santos et al. [36] treated several cell lines infected with SARS-CoV-2 with CBD and proprietary mixtures of terpenes derived in part from *C. sativa*. They found that CBD as well as the terpene mixtures inhibited viral replication and that the combination of both had an additive effect (Table 1). Although it was suggested that CBD prevented the release of proinflammatory cytokines by functioning as a partial agonist of the cannabinoid receptor-2, no cytokine measurements were reported.

By measuring mRNA changes in lung alveolar A549 cells that had been stably overexpressed with human ACE2 and infected by SARS-CoV-2 with or without CBD treatment (2.5 µM), Nguyen et al. [27] concluded that CBD can inhibit the stimulation of cytokines associated with COVID-19 and that a mechanism of action might include activation of serine/threonine-protein kinase/endoribonuclease inositol-requiring enzyme 1α. In a review of anti-inflammatory effects of cannabinoids, Janecki et al. [37] observed that activation of the cannabinoid receptor-1 and especially cannabinoid receptor-2 by cannabinoids could inhibit the release of proinflammatory cytokines as well as stimulate production of anti-inflammatory cytokines (Figure 2). Note that Δ^9^-THC has high affinity for both cannabinoid receptor-1 and cannabinoid receptor-2 [38].

Using human monocytic THP-1 cells and primary human macrophages infected with HIV, Tomer et al. [39] measured mRNA changes induced by CBD treatment (10 µg/ml). CBD was found to decrease interferon-regulated gene expression while increasing autophagy (Table 1 and Figure 2). Interferon-regulated gene expression decreased due to attenuated cGAS-STING-mediated activation and was probably responsible for an increase in HIV RNA expression 24 h post-infection. However, up-regulation of the autophagy receptor p62/SQSTM1 resulted in reduced HIV viral spread during 8-day incubations.

### CBDA, CBGA, and Δ^9^-THCA-A

Using affinity-selection mass spectrometry to identify low molecular weight ligands to the SARS-CoV-2 spike protein that might block binding to human ACE2 [40], van Breemen et al. [41] identified CBDA, CBGA, and Δ^9^-THCA-A as high affinity ligands to the spike protein S1 subunit. Molecular docking experiments indicated that all three cannabinoids bound orthosterically to the spike protein while CBGA also bound to an allosteric site. CBGA and CBDA bound to the SARS-CoV-2 spike protein S1 subunit with *K*d values of 19.8 ± 2.7 and 5.6 ± 2.2 μM, respectively. CBGA and CBDA prevented infection of human epithelial cells by a pseudovirus expressing the SARS-CoV-2 spike protein, prevented entry of live SARS-CoV-2 into cells, and were equally effective against the SARS-CoV-2 alpha variant B.1.1.7 and the beta variant B.1.351 (Table 1; Figure 2).

In a study of minor cannabinoid inhibitors of 3CLpro, the second most potent inhibitor reported by Liu et al. [23] was CBGA with an IC_50_ value of 14.40 μM (Table 1). Their preliminary structure and activity relationship study suggested that carboxylation status influenced activity. Decarboxylation of CBGA to form CBG reduced the IC_50_ for 3CLpro by more than 10-fold.

### Cannabichromanon, cannabinolic acid, cannabinol, and cannabicyclolic acid

Using molecular docking, Altyar et al. [42] screened a library of 45 cannabinoids for binding to the active site of the SARS-CoV-2 protease 3CLpro. Although the inhibitors Δ^9^-tetrahydrocannabibutol, CBD, and CBGA were included in the library, the best ligands were predicted to be cannabichromanon followed by cannabinolic acid and cannabinol with Δ*G* values of −33.63, −23.24, and −21.60 kcal/mol, respectively (Table 1 and Figure 2). These cannabinoids were also predicted to be the best ligands for ACE2 with Δ*G*  values of −41.77, −31.34, and −30.36 kcal/mol, respectively. Because no functional enzyme assays or antiviral assays were carried out, it remains unknown how effectively these cannabinoids inhibit 3CLpro or ACE2 and if they have anti-SARS-CoV-2 efficacy *in vivo*. Also in this *in silico* study, Altyar et al. [42] predicted that cannabichromanon, cannabinolic acid, and cannabicyclolic acid (Figure 1) would be the highest affinity ligands for the active site of the other SARS-CoV-2 protease, PLpro, with Δ*G*  values of −28.36, −22.81, and −19.89 kcal/mol (Figure 2). Again, no enzyme assays or antiviral activity experiments were reported using these cannabinoids.

### Cannabivarin and Δ^8^-THCA-A

Using molecular modeling, El Ouafi et al. [43] predicted that cannabivarin and Δ^8^-THCA-A should bind to the human cell receptor ACE2 with docking scores of −8.3, −8.0, and −8.3 kcal/mol, respectively (Table 1 and Figure 2). Although these data suggest that cannabivarin and Δ^8^-THC might inhibit ACE2 even better than Δ^9^-THC or CBD, no laboratory measurements were carried out to confirm inhibition of ACE2 or to determine antiviral activity. However, this *in silico* study suggests that minor cannabinoids might be highly effective as antiviral agents and warrant further investigation.

## Discussion

During most of the last century, legal restrictions impeded cultivation of hemp, isolation of cannabinoids, and research on the antiviral properties of cannabinoids and other hemp secondary metabolites. Furthermore, the discovery of antiviral agents showed little progress until the discovery of antiviral targets guided by molecular biology in the 1980s. Since then, cannabinoids have been shown to have activity against herpes viruses, which are DNA-type viruses, as well as the RNA-type viruses SARS-COV-2 and HIV/SIV.

The most studied antiviral cannabinoids to date have been CBD and Δ^9^-THC, which have been approved as drugs by the FDA for unrelated pharmacological activities. These two cannabinoids function as antiviral agents through multiple mechanisms of action, some of which overlap such as inhibition of the SARS-CoV-2 main protease 3CLpro and inhibition of ACE2, which is the human cell receptor for SARS-CoV-2 (Table 1 and Figure 2). CBD and Δ^9^-THC also have anti-inflammatory activities that can help suppress the proinflammatory effects of SARS-CoV-2 and HIV/SIV (Table 1). Other cannabinoids have recently been shown to have antiviral activities that include some unique mechanisms of action. For example, CBDA and CBGA can prevent cell entry and infection by SARS-CoV-2 (Table 1 and Figure 2). Research on the antiviral activities of the more than 100 less abundant cannabinoids is just beginning, and there is potential to discover even more potent antiviral agents among these unique chemical structures.

Importantly, clinical trials are needed to explore the safety and efficacy of antiviral cannabinoids. Based on the multiplicity of active cannabinoids acting by different mechanisms of action, combinations of cannabinoids should be explored for activity. Combination therapy has become the mainstay of HIV antiretroviral therapy due to the superior activity of drug mixtures that act by complementary mechanisms of action [44]. Furthermore, combination antiviral therapy can slow the development of resistant virus strains [45]. Phase 1 clinical trials of antiviral cannabinoids are needed to establish appropriate dosing routes, such as per oral or sublingual, as well as appropriate dosage levels and dosing frequencies to reach and sustain therapeutically active plasma concentrations. Combinations of antiviral cannabinoids should also be optimized to account for possible cannabinoid–cannabinoid interactions such as inhibition or induction of drug metabolizing enzymes and transporters [46]. Clinical trials are also needed to explore cannabinoid-drug pharmacokinetic interactions to help ensure their safe and effective use.

Appropriately designed Phase 2 clinical trials of antiviral cannabinoids are required to establish safety and efficacy. Unlike most previous clinical trials of antiviral cannabinoids, these Phase 2 trials should be prospective, placebo controlled, randomized, double-blind, and include enough participants to provide conclusive results. Most of all, future Phase 2 clinical trials should utilize dosages selected to achieve efficacious plasma concentrations. The use of subtherapeutic dosages of CBD caused the failure of the otherwise well-designed Phase 2 clinical trial with COVID-19 patients by Crippa et al. [30]. Although most antiviral cannabinoids have shown individual activities in the low micromolar range, combinations of cannabinoids acting through complementary mechanisms of action might show synergistic effects that might be efficacious at lower concentrations. Synergy among cannabinoids known as an entourage effect has already been established for pharmacological activities such as pain management [47].

Antiviral activities of some of the most abundant cannabinoids have been documented *in silico*, *in vitro*, and *in vivo*. Studies of the antiviral activities of the more than 100 less abundant cannabinoids are still needed as are carefully designed clinical trials. Based on the preclinical evidence of antiviral activity as well as oral bioavailability and long history of safe human use of cannabinoids individually or as mixtures, multiple clinical studies of antiviral cannabinoid safety and efficacy are in progress worldwide using CBD [48] and Δ^9^-THC [49]. and additional studies will certainly follow.

## Data Availability

This review article contains references to all the published scientific literature and web resources that were consulted.

## Abbreviations

Δ^9^-THC: Δ^9^-tetrahydrocannabinol
Δ^9^-THCA-A: Δ^9^-tetrahydrocannabinolic acid-A
ACE2: angiotensin converting enzyme 2
CBD: cannabidiol
CBDA: cannabidiolic acid
CBGA: cannabigerolic acid
HIV: human immunodeficiency virus
IL: interleukin
LPS: lipopolysaccharide
SARS-CoV-2: severe acute respiratory syndrome coronavirus-2
SIV: simian immunodeficiency virus
TNF-α: tumor necrosis factor-α
